# Clinical Insights Into Addressing Constricted Maxillary Arch in Angle’s Class II Malocclusion: A Case Report

**DOI:** 10.7759/cureus.55798

**Published:** 2024-03-08

**Authors:** Dhwani Suchak, Ranjit Kamble, Pallavi Daigavane, Nikhil Kumar, Nishu Agarwal, Lovely Bharti

**Affiliations:** 1 Orthodontics and Dentofacial Orthopedics, Sharad Pawar Dental College and Hospital, Datta Meghe Institute of Higher Education & Research, Wardha, IND; 2 Orthodontics and Dentofacial Orthopedics, Kusum Devi Sunderlal Dugar Jain Dental College & Hospital, Kolkata, IND

**Keywords:** intermolar width, palatal expansion, posterior crossbite, orthodontic forces, slow maxillary expansion

## Abstract

The transverse dimension, often overlooked in orthodontics, plays a crucial role in malocclusions, affecting not only occlusion in that dimension but also sagittal and vertical dimensions. Posterior crossbites, indicative of transverse maxillary issues, are commonly addressed through palatal expansion. This case report explores the clinical insights into addressing a constricted maxillary arch in Angle's Class II malocclusion using a nickel-titanium (NiTi) expander.

The NiTi expander provides constant and optimal expansion forces by incorporating a temperature-activated NiTi alloy. A 16-year-old male with irregularly placed teeth, high palatal vault, and posterior crossbite underwent treatment involving NiTi expander usage for maxillary expansion. The case presentation details the patient's journey, starting with upper arch bonding and expansion, then lower arch bonding, and concluding with complete leveling and alignment without extractions.

The presented case demonstrates successful correction of a constricted maxillary arch, specifically in the canine and molar regions, utilizing the NiTi expander. The observed increase in intermolar width aligns with previous studies, showcasing the effectiveness of slow maxillary expansion. This article contributes valuable clinical insights into addressing transverse maxillary issues, emphasizing the importance of careful consideration in choosing the appropriate expansion method for optimal results.

## Introduction

In the field of orthodontics, the transverse plane, one of the three spatial dimensions along with sagittal and vertical, is the least explored. Timely intervention is crucial for malocclusions in the transverse dimension, as they affect occlusion in that dimension and have implications for sagittal and vertical dimensions. The significance of the transverse dimension lies in its limited growth, early cessation of growth, and often reaching maturity by the time patients are observed. Posterior crossbite usually indicates transverse issues in the maxilla, and palatal expansion is a widely used and established method for correcting crossbites in children and adolescents [1]. Palatal expansion usually uses a combination of both orthodontic and orthopedic forces [2]. Strong forces can lead to the separation of the sutures of the maxilla as well as those surrounding it. The amount of expressed orthodontic or orthopedic forces depends more or less on the patient's age [3,4]. Increases in arch width obtained through slow palatal expansion procedures are generally thought to result in an orthodontic response with little if any, orthopedic component [5-7].

The nickel-titanium (NiTi) expander delivers constant and optimum expansion forces [8]. The NiTi expander can be used concurrently with fixed appliances for expansion to continue with the leveling of the rest of the arch. The core component comprises a temperature-activated NiTi alloy, whereas the remainder of the apparatus is made of stainless steel (SS). The transition temperature for the NiTi part of the expander is 94°F; hence, at normal room temperature, it is rigid while inserting. Chilling the expander causes the central component to soften, making it easy to manipulate. Once inserted, the temperature of the expander rises to the temperature inside the oral cavity, stiffens, and begins to revert to its initial form. A 3mm increase in expansion delivers 350g of force [8,9].

## Case presentation

**Figure 1:**
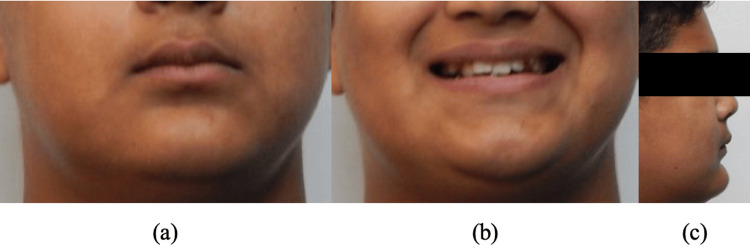
Pre-treatment extra-oral images: (a) Frontal (b) Frontal smiling (c) Profile

**Figure 2:**
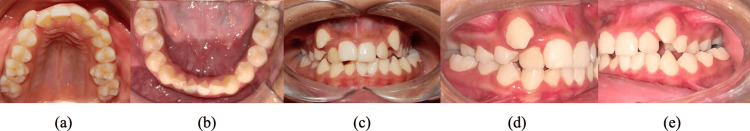
Pre-treatment intra-oral images: (a) Maxillary occlusal photograph (b) Mandibular occlusal photograph (c) Anterior in occlusion (d) Right molar in occlusion (e) Left molar in occlusion

**Figure 3:**
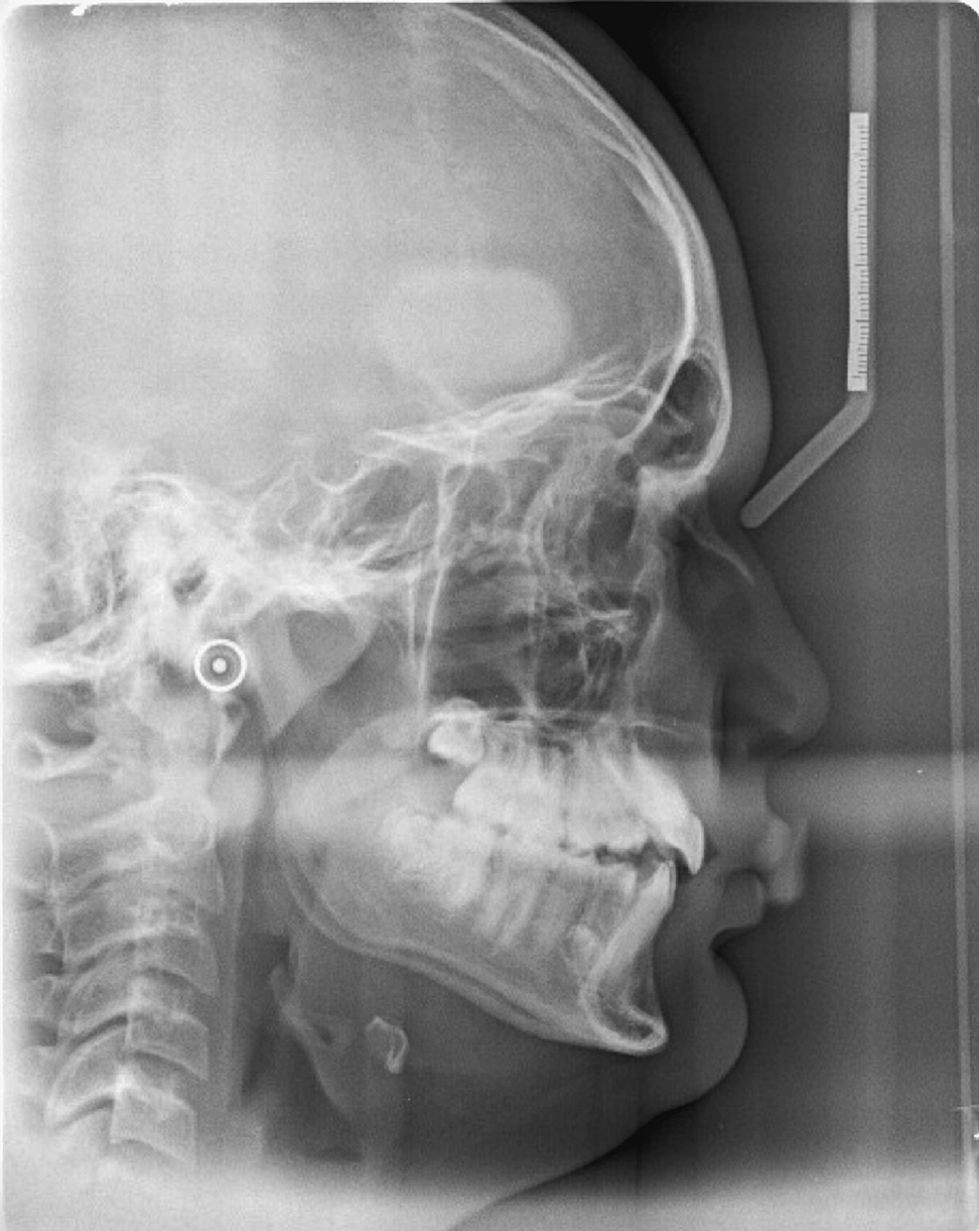
Pre-treatment lateral cephalogram

A male patient, aged 16 years, reported a chief complaint of irregularly placed teeth. The extraoral examination revealed a straight profile with a deep mento labial sulcus and an obtuse nasolabial angle (Figure 1). The intra-oral examination of the patient revealed buccally placed canines, high palatal vault, and posterior crossbite. The mandibular anterior region revealed crowding. Angle's Class II malocclusion was seen on the right side, and Angle's Class I malocclusion was on the left side (Figure 2). The cephalometric analysis showed skeletal Class I bases, proclined upper incisors, upright lower incisors, and an obtuse nasolabial angle (Figure 3). The analysis of the study models revealed a constricted maxillary arch in both the canine and the molar region.

**Figure 4:**
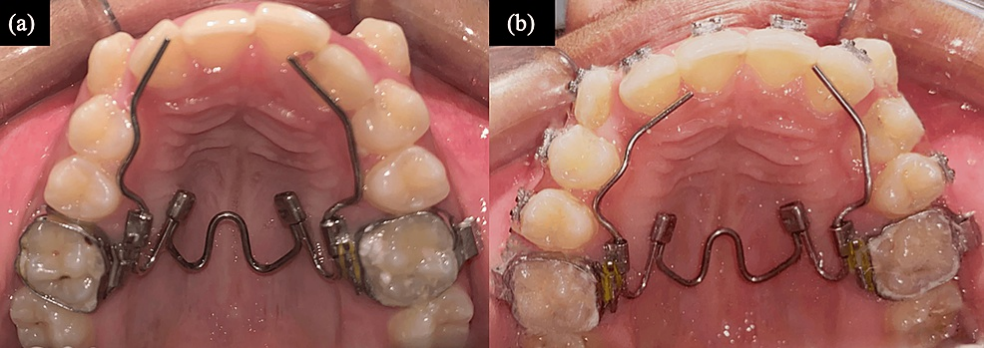
With appliance photographs (a) Pre-expansion (b) Post-expansion

**Figure 5:**
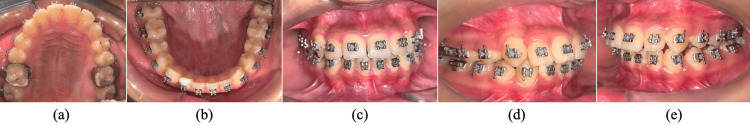
Post-treatment intra-oral images: (a) Maxillary occlusal photograph (b) Mandibular occlusal photograph (c) Anterior in occlusion (d) Right molar in occlusion (e) Left molar in occlusion

**Figure 6:**
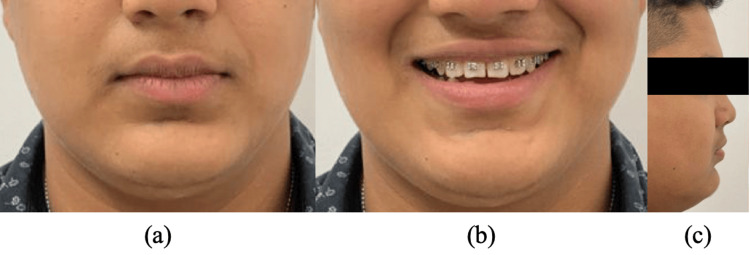
Post-treatment extra-oral images: (a) Frontal (b) Frontal smiling (c) Profile

To correct the posterior crossbite, expansion of the upper arch was considered. The case started with the bonding of the upper arch and expansion. A slow type of expander, i.e., NiTi expander, was used for expansion. Expansion was continued for six months (Figure 4). Following the correction of the posterior crossbite, bonding of the lower arch was done. Complete leveling and alignment of both arches were done, and the treatment was completed without extractions (Figure 5). The post-treatment extra-oral photographs revealed a straight profile, competent lips, and an obtuse nasolabial angle (Figure 6). The total treatment lasted for 18 months.

## Discussion

The recognition of a narrow maxilla dates back thousands of years [10]. Approximately 25-30% of patients undergoing orthodontic treatment can benefit from maxillary expansion [11]. The transverse maxillary expansion serves not only to remedy posterior crossbite, leading to the advancement of the maxilla but also to generate extra space in the arch, facilitating the alignment of overcrowded permanent teeth. This expansion process can be categorized into two types: rapid maxillary expansion (RME) and slow maxillary expansion (SME). SME appliances exert mild forces, initiating the opening of the suture at a pace similar to the maximum rate of bone growth.

Bell postulated that the rate at which midpalatal suture separates in SME results in a more manageable response compared to the potentially destructive forces produced by RME. This preserves tissue integrity, ensures greater stability, and reduces the likelihood of relapse [12]. After a thorough clinical evaluation, SME was chosen as the treatment for our patient.

With a posterior crossbite and constricted maxilla, our patient experienced correction and an observable change in the intermolar width. Changes in intermolar width reflect the total dentoalveolar expansion achieved by the appliance. In our case, intermolar width increased, aligning with findings from other studies [9,13-16]. About 10mm of increase in intermolar width is seen in RME, and in SME, up to 8mm increase can be seen [17]. Various studies have reported increases in intermolar width, such as 5.3 mm by Bell and Lecompte [13], 5.88 mm by Frank and Engel [14], 6.26 mm by Ciambotti et al. [15] using NiTi expanders, 8.5 mm by Karaman [16] and an increase reported by Marzban and Nanda [9].

The NiTi expander's SS extension accounts for the expansion at the canine and molar regions, which were 7mm and 5mm, respectively. This increase in intercanine width aligns with the findings of Bell and Lecompte [13], Frank and Engel [14], and Ferrario et al. [18].

## Conclusions

The presented case report sheds light on the effective clinical management of Angle's Class II malocclusion with a constricted maxillary arch using a nickel-titanium (NiTi) expander. The emphasis on transverse maxillary expansion is crucial, considering its impact on occlusion in multiple dimensions. Utilizing a slow maxillary expansion (SME) approach with the NiTi expander proved to be a successful intervention, demonstrating observable changes in intermolar width and achieving the desired correction. The presented case contributes valuable insights into the clinical application of the NiTi expander, showcasing its effectiveness in addressing specific challenges associated with a constricted maxillary arch. The observed increase in intermolar width aligns with previous studies, reinforcing the importance of careful consideration in selecting the appropriate expansion method.

Overall, this case report is a valuable resource for orthodontic practitioners, providing clinical perspectives on managing transverse maxillary issues in Angle's Class II malocclusion. The successful outcomes in this case underscore the significance of timely intervention, precise treatment planning, and the integration of advanced orthodontic appliances for optimal results in orthodontic practice.
